# FP-GCN: Frequency Pyramid Graph Convolutional Network for Enhancing Pathological Gait Classification

**DOI:** 10.3390/s24113352

**Published:** 2024-05-23

**Authors:** Xiaoheng Zhao, Jia Li, Chunsheng Hua

**Affiliations:** 1 Institute of Intelligent Robots and Pattern Recognition, School of Cyber Science and Engineering, Liaoning University, Shenyang 110036, China; 2 Department of Endocrinology and Metabolism, The Fourth Affiliated Hospital of China Medical University, Shenyang 110032, China

**Keywords:** pathological gait recognition, skeleton-based gait analysis, deep-learning, graph convolutional network

## Abstract

Gait, a manifestation of one’s walking pattern, intricately reflects the harmonious interplay of various bodily systems, offering valuable insights into an individual’s health status. However, the current study has shortcomings in the extraction of temporal and spatial dependencies in joint motion, resulting in inefficiencies in pathological gait classification. In this paper, we propose a Frequency Pyramid Graph Convolutional Network (FP-GCN), advocating to complement temporal analysis and further enhance spatial feature extraction. specifically, a spectral decomposition component is adopted to extract gait data with different time frames, which can enhance the detection of rhythmic patterns and velocity variations in human gait and allow a detailed analysis of the temporal features. Furthermore, a novel pyramidal feature extraction approach is developed to analyze the inter-sensor dependencies, which can integrate features from different pathways, enhancing both temporal and spatial feature extraction. Our experimentation on diverse datasets demonstrates the effectiveness of our approach. Notably, FP-GCN achieves an impressive accuracy of 98.78% on public datasets and 96.54% on proprietary data, surpassing existing methodologies and underscoring its potential for advancing pathological gait classification. In summary, our innovative FP-GCN contributes to advancing feature extraction and pathological gait recognition, which may offer potential advancements in healthcare provisions, especially in regions with limited access to medical resources and in home-care environments. This work lays the foundation for further exploration and underscores the importance of remote health monitoring, diagnosis, and personalized interventions.

## 1. Introduction

Gait analysis is a fundamental area of study that elucidates the intricate coordination among various bodily systems, including muscles, bones and nerves, as well as the cardiovascular and respiratory systems. This sophisticated interplay is indispensable for achieving stable and efficient locomotion. However, disruptions stemming from musculoskeletal injuries, neurological disorders, or systemic diseases can induce pathological gaits, characterized by abnormal walking patterns that impede mobility. Accurate detection of these disturbances through gait classification is pivotal for early diagnosis and monitoring progress in both medical and engineering domains.

Traditionally, gait classification has relied on non-visual sensor-based methods employing specialized equipment such as accelerometers to capture biomechanical signals during walking. Despite their high accuracy, these methods often entail limitations. The equipment can be costly, susceptible to environmental interference, and necessitate controlled testing environments, thereby restricting accessibility in medically underserved regions and home settings. Video-based methods present a promising alternative to these conventional approaches. By utilizing readily available cameras, video-based methods offer a more pragmatic and potentially less intrusive solution for gait analysis. Additionally, they introduce opportunities for applications like remote gait assessment, which could further enhance accessibility and convenience.

This paper presents a novel video-based approach for pathological gait classification. Our main innovation lies in the Frequency Domain Graph Convolutional Network (F-GCN). F-GCN addresses the challenge of feature extraction by concurrently analyzing both temporal and spatial information in the gait data. This integrated analysis yields a more comprehensive representation of walking patterns, resulting in more accurate classification. To augment the model’s ability to capture spatial dependencies within these enriched features, we introduce the Pyramid Graph Convolutional Network (P-GCN). P-GCN efficiently utilizes the feature representation generated by F-GCN, ultimately leading to improved pathological gait classification. This novel approach is particularly advantageous for medically underserved and home monitoring, facilitating enhanced detection and management of gait abnormalities. Our comprehensive research yields the following key innovations:

- The F-GCN significantly enhances feature extraction in gait analysis by capturing temporal dependencies within the gait cycle using frequency domain principles. This enables a more thorough analysis of walking patterns, leading to improved accuracy in pathological gait classification.
- The P-GCN refines spatial feature extraction, leveraging the richer features created by F-GCN. This multi-scale and multi-space partitioning approach further augments the accuracy of pathological gait classification.
- Multiple dataset experiments showcase the effectiveness of our methods and the superiority of our proposed approach.

## 2. Related Works

Over the decades, pathological gait classification has seen significant advancements and can be approached through two main categories: non-visual sensor-based methods and video-based methods.

### 2.1. Non-Visual Sensor-Based Methods

**Figure 1:**
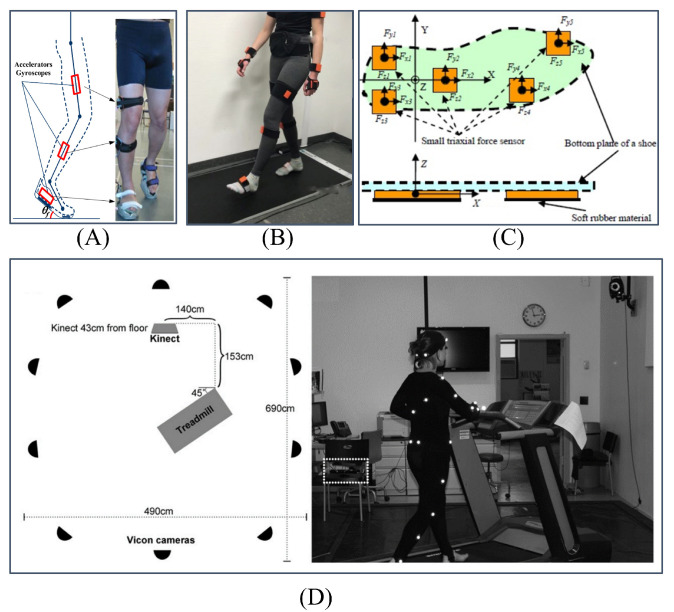
Some examples of pathological gait classification approaches: (**A**) Accelerometer [4] could be arranged on a patient’s leg to get gait parameters; (**B**) Force plate method [5] could collect the ground reaction forces to analyse the gait; (**C**) Fabric sensors [6] could be applied as smart insole to collect the data from the human foot; (**D**) in [7], 3D Vicon motion capture and Kinect camera are combined for gait analysis.

Non-visual sensor-based methods offer a valuable approach to gait analysis. As shown in Figure 1, these methods leverage sensors like accelerometers [1], round reaction force [2], and force plates [3], to capture biomechanical signals during walking, such as acceleration, ground reaction force, and pressure distribution. By analyzing these signals, researchers can assess various gait characteristics and patterns. However, their application is limited by several factors. These methods often require expensive equipment like specialized sensors, restricting their accessibility in resource-constrained settings. Additionally, controlled environments like laboratories are necessary to minimize the influence of external factors on sensor data. Prolonged wear of these sensors can also cause discomfort for some patients. These limitations hinder the widespread adoption of non-visual methods, particularly in medically underserved regions where access to advanced gait analysis equipment might be limited. Furthermore, the requirement for controlled settings makes them less suitable for home-based gait monitoring. Its overarching goal is to facilitate the timely identification of pathological gait, thereby alleviating the strain on healthcare resources and minimizing patient suffering.

### 2.2. Video-Based Methods

Early video-based approaches for gait analysis relied on silhouette analysis and Gait Energy Images (GEI). Albuquerque et al. [8] introduced a machine learning approach utilizing Long Short-Term Memory (LSTM) networks for pathological gait classification based on silhouette frames, refs. [9,10] employed gait silhouettes and GEI to aid diagnosis pathological gait. Nghiem et al. [11] presented an approach through the analysis of symmetry in leg movements. However, these silhouette-based methods often require high-quality video inputs and precise image segmentation, imposing stringent hardware and equipment requirements.Silhouette-based methods lack the resolution to capture the nuances of joint movements, limiting their ability to provide finer-grained information about gait dynamics.

Additionally, due to the complexity and diversity of pathological gait data, traditional algorithms may not suffice for the classification of all pathological gait patterns. With the advent of depth cameras like Kinect, researchers have shifted towards using skeletal joint points for pathological gait analysis. Bei et al. [12] proposed a machine-learning approach that extracts leg swing features using depth cameras for motion disorder detection. Gu et al. [13] introduced a Cross-Domain learning method that captures geometric information of the lower limbs using depth cameras for pathological gait classification. The rapid development of deep learning and image processing technologies has propelled the utilization of GCNs in the field of action classification. Researchers have begun harnessing the power of GCNs to tackle pathological gait classification challenges. For instance, Zeng et al. [14] introduced Slow-Fast GCN to quantify Parkinsonian gait by detecting bilateral asymmetry and gait features. Tian et al. [15] proposed AGS-GCN, which introduced a novel joint partitioning strategy and attention mechanism for pathological gait classification. Jun et al. [16] implemented a bidirectional gated recurrent network algorithm for abnormal gait video classification. Jun et al. [17] explored a multi-input spatio-temporal graph convolutional network using RGB-D cameras to extract skeletal joint points for pathological gait classification. RGB-D cameras have limitations in terms of sensitivity to lighting conditions and are often constrained to indoor or close-range environments. Notably, studies by Dingenen et al. [18] and Stenum et al. [19] demonstrated the accuracy of 2D data in analyzing human gait, emphasizing its effectiveness compared to 3D data. Visual-based approaches in pathological gait analysis have evolved from silhouette-centric techniques to depth cameras like Kinect, enabling more precise skeletal joint point analysis. While recent advancements incorporate graph convolutional networks (GCNs) for action recognition, challenges persist with RGB-D camera limitations, leading to alternative methods such as multi-camera setups and 2D skeletal point analysis using visible cameras. The field continues to seek innovative solutions for comprehensive pathological gait analysis.

### 2.3. Comparative Analysis: Non-Visual and Visual Methods

Although the wearable-sensor-based systems could directly capture the biomechanical signals from patients, they are not suitable for normal daily health monitoring and usually suffer from the following problems: (1) requiring cooperation from patients which means their movements are not in their natural state; (2) sensitive to the noise produced by the electronic sensors and involuntary movement of human; (3) requiring special equipment and not suitable to be applied at home; (4) almost impossible to be worn for long time; (5) expensive costs, etc. Due to all these reasons, with the rapid progress in computer vision and deep learning algorithms, the proposed camera-based method is more suitable for the daily health monitoring of patients at home, because the vision-based pathological gait recognition algorithm doesn’t require cooperation from patients and other special sensors.

Furthermore, compared with the wearable-sensor-based methods which are usually restricted to special body parts, the proposed method is based on the touchless video analytical method, which can capture the movements of the entire body, including the head, torso, arms, and legs as well as their motion features. Such features could include: the symmetry and coordination of human limbs, joint angles, step length/frequency, torso inclination, etc. Usually, it is quite difficult for the motion-sensor-based method to capture and analyze those features simultaneously. Such ability to observe the movements of the entire body is vital for diagnosing neurological disorders such as stroke and Parkinson’s disease.

## 3. Method

### 3.1. Overview

**Figure 2:**
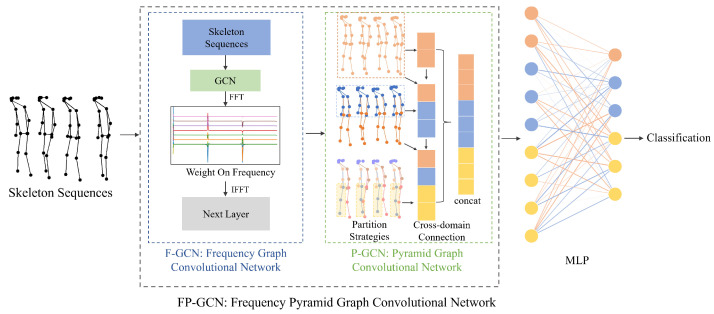
Overview of the proposed Frequency Pyramid Graph Convolutional Network. The F-GCN improves gait analysis by efficiently capturing temporal dependencies through frequency domain. And the P-GCN enhances spatial feature extraction by employing multi-scale and multi-space partitioning. boosting the accuracy of classification.

Figure 2 illustrates the detail structure of FP-GCN, a deep learning method designed for pathological gait classification. FP-GCN addresses a critical challenge: the significant variations in walking speed observed across different gait pathologies. Traditional methods, like Temporal Convolutional Networks (TCNs), rely on fixed-window approaches that struggle to adapt to these variations. To overcome this limitation, FP-GCN incorporates two specialized modules:

F-GCN: This module tackles the challenge of variable temporal patterns. It operates in the frequency domain, effectively capturing and weighting the temporal dependencies within gait data.

P-GCN: This module focuses on capturing the spatial relationships between different body joints. Motor impairments often involve complex interactions across multiple body systems. P-GCN’s unique capability for multi-scale and multi-regional spatial segmentation allows it to optimize the extraction of spatial features, leading to more accurate gait recognition.

The effectiveness of FP-GCN is validated through experiments on both publicly available datasets and a custom dataset. These experiments demonstrate that FP-GCN achieves significantly better recognition results compared to the conventional ST-GCN approach.

### 3.2. Frequency Graph Convolutional Network

**Figure 3:**
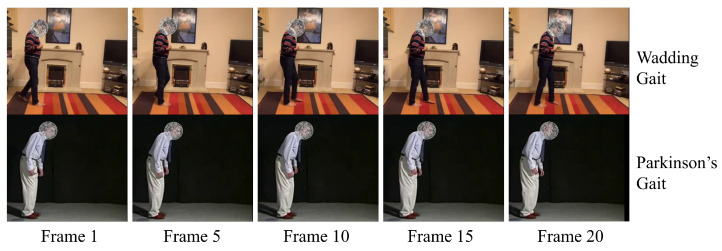
The illustration of walking speed variation between two pathological gaits. Since different organs of the patient are damaged by diverse diseases, the walking cycles of two pathological gaits could be changed greatly.

Gait analysis, influenced by factors including age, physical condition, and environmental aspects, exhibits considerable variability. Diseases can further exacerbate these variations, introducing abnormalities detectable in walking patterns. Figure 3 contrasts walking speeds in various pathological gaits, illustrating changes in speeds for conditions such as Parkinson’s and unsteady gaits. Unlike traditional Time Convolutional Network (TCN) methods, which are limited by fixed time windows, this study proposes the Frequency Graph Convolutional Network (F-GCN). F-GCN improves pathological gait analysis accuracy by extracting temporal features in the frequency domain for more adaptable and precise processing.

**Figure 4:**
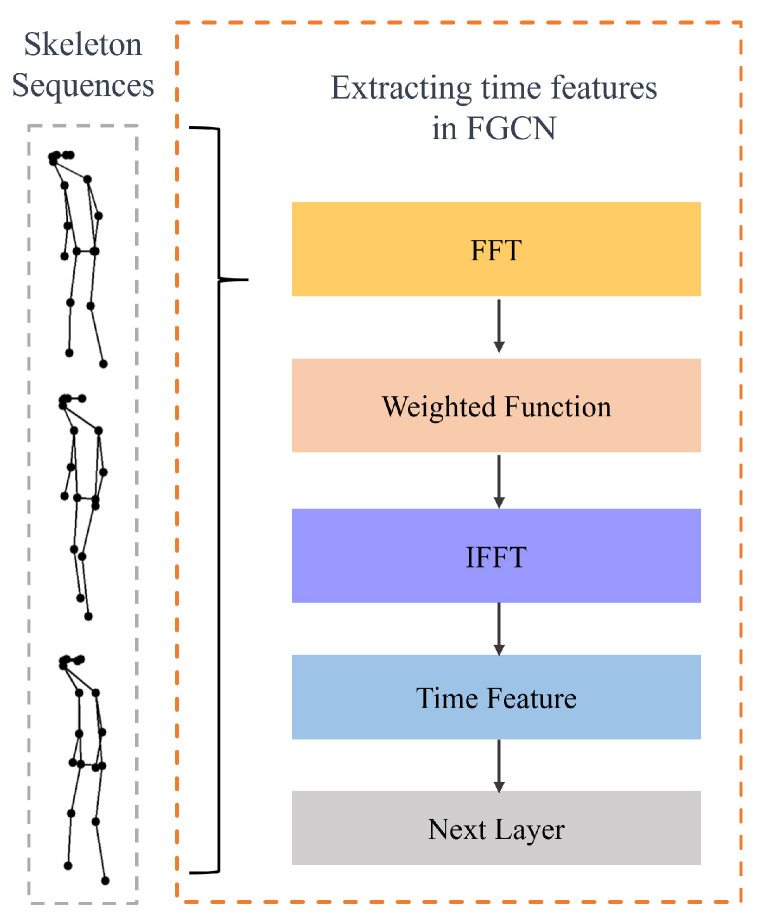
The Frequency Graph Convolutional Network (F-GCN) considers the entire time feature and operates in the frequency domain, enabling it to effectively analyze temporal patterns and dependencies.

The computation of Time Convolutional Networks (TCNs), detailed in Equation (1), inadequately captures temporal variations and differences. By converting data into the frequency domain using two-dimensional fast Fourier transform (2DFFT) as shown in Equation (3), and weighting the spatial and temporal features based on training weights from graph convolution, the F-GCN offers an enhanced feature representation. This integrated approach allows the model to better discern spatial and temporal patterns, improving its performance in gait analysis. Figure 4 depicts the F-GCN model, highlighting its ability to minimize the noise introduced by human skeleton prediction algorithms and to capture the intrinsic periodicity of human gait. The F-GCN’s consideration of both spatial and temporal characteristics promises a more comprehensive understanding of gait features, beneficial for applications in healthcare and rehabilitation.

(1) $$Y_{i}^{\left(t+1\right)}=\sum_{j=0}^{N_{j}}\sum_{\tau =0}^{sd}W^{t}*X_{j}^{t+\tau },$$

where the output $Y_{i}^{\left(t+1\right)}$ of the time convolutional layer represents the features of node *i* at frame t+1. $N_{j}$ denotes the set of neighboring nodes for node *i*. $W_{t}$ represents the convolutional kernel for the current time step, which is a set of learnable parameters. And $X_{j}^{t}$ is the feature of neighboring node *j* at the current frame *t* up to the time stride sd.

Although TCNs perform well in specific contexts, their fixed time window analysis hinders the accurate capture of variations in gait data. Addressing this limitation, the present study introduces the Frequency Graph Convolutional Network (F-GCN), which utilizes the frequency domain for temporal feature extraction. This approach affords greater flexibility and efficacy in the analysis, enhancing the accuracy of pathological gait assessment.

Weighted Function On Spatial: the Spatial Graph Network (SGN) determines the node features $X_{i}^{\prime }$ and the edge weights $W_{E}$, as formalized in Equation (2).

(2) $$\begin{array}{cc} X_{i}^{\prime } & =\mu (\frac{1}{d_{i}}\sum_{j=1}^{E_{i}}W_{E_{\left(j\right)}}*X_{j}),\\ X^{\prime } & =\{X_{0}^{\prime },X_{1}^{\prime },X_{2}^{\prime }\ldots \ldots X_{j}^{\prime }\},j\in V, \end{array}$$

where $X_{i}^{\prime }$ represents the features extracted by the model at node *i*, μ(·) denotes the activation function. $E_{j}$ represents the set of edges for the current node *i*. $d_{i}$ denotes the degree of the node *i*, used here for normalizing. $W_{E_{\left(j\right)}}$ is a learnable weight of the *j*-th edge.

Weighted Function On Temporary: The model extracts temporal features in the frequency domain through the following calculation process: employing a two-dimensional fast Fourier transform (2*DFFT*) on the temporal dimensions to convert the data into the frequency domain. To ensure scale consistency between the Fourier transform and its inverse, orthogonal normalization is conducted during the computation. As shown in Equation (3), in this step, the model utilizes a two-dimensional fast Fourier transform (2*DFFT*) on the temporal dimensions to transform the data into the frequency domain. The orthogonal normalization is carried out to maintain scale consistency between the Fourier transform and its inverse.

(3) $$\begin{array}{cc} X_{f}\left[K_{S},K^{T}\right] & =2DFFT\left(X^{\prime }\right)=2DFFT\left(X_{S},X^{T}\right)\\ \; & =\frac{\sum_{s=0}^{S}\sum_{t=0}^{T}\left(X_{s}^{\prime },X^{\prime t}\right)}{\sqrt{ST}}*e^{j2\pi (\frac{K_{S}X_{S}^{\prime }}{S}+\frac{K^{T}\cdot X^{\prime T}}{T})}, \end{array}$$

where $X_{f}$ represents the transformed features in the frequency domain. $K_{S}$ and $K^{T}$ are the eigenvalues of the input features in the spatial and temporal dimensions. $X_{S}$ and $X^{T}$ are the eigenvalues of the input in the spatial and temporal dimension. Here, *S* is the length of the spatial dimension, transformed from the input dimension C·V, and *T* is the length of the temporal dimension. $K_{S}$ denotes the eigenvalues on the discrete spatial coordinates in the frequency domain, with a length of S−1 and $K^{T}$ represents the eigenvalues on the discrete time coordinates in the frequency domain, with a length of (⌈T/2+1⌉), *e* is the natural logarithm, and *j* represents the imaginary part.

After transforming the data into the frequency domain, the next step involves weighting in both the temporal and spatial frequency domains based on the training weights obtained from graph convolution. As shown in Equation (4). This process is crucial for incorporating the learned spatial and temporal dependencies into the model. The weighted features contribute to a more comprehensive representation, capturing both spatial and temporal aspects of the data. This integration enhances the model’s ability to discern patterns and relationships, ultimately improving its performance in analyzing the given information.

(4) $$\begin{array}{cc} X_{f}^{\prime }\left[K_{S}^{\prime },K^{\prime T}\right] & =(W_{A}\left(K_{S}\right),W_{F}\left(K^{T}\right))\\ \; & =\left(\left(\frac{1}{S}\sum_{s=0}^{S}\sum_{i=0}^{W_{E}}\theta \left(W_{E_{\left(i\right)}}\cdot W_{A_{\left(s\right)}}\cdot K_{S}\right)\right),\;\left(\frac{1}{\lceil T/2\rceil +1}\sum_{t=0}^{\lceil T/2\rceil +1}\theta \left(W_{F}^{t}\cdot K^{T}\right)\right)\right), \end{array}$$

where $X_{f}^{\prime }$ represents the features weighted in the frequency domain. $W_{E}$ represents the relationship weights mentioned in Equation (2), $W_{A}$ and $W_{F}$ are trainable weights, and θ(·) denotes the LeakyReLU activation function. The terms $\frac{1}{S}$ and $\frac{1}{\lceil T/2\rceil +1}$ are used for normalization along the spatial and frequency dimensions. Furthermore, because of the symmetry of the frequency spectrum, only the positive and zero frequencies need to be considered, which extends to T/2+1.

Lastly, employing an inverse Fourier transform, F-GCN recalibrates the features to output $Y_{FGCN}$ as delineated in subsequent equations. With frequency domain weighting and original space restoration, F-GCN effectively diminishes computational noise from skeleton point predictions and capitalizes on the inherent periodicity of human gait to precisely extract gait features, while integrating spatial information to provide a holistic feature representation that encompasses both frequency domain and spatial properties.

(5) $$\begin{array}{cc} Y_{FGCN}\left[Y_{S},Y^{T}\right] & =2DIFT\left(X_{f}^{\prime }\right)=2DIFT\left(K_{S}^{\prime },K^{\prime T}\right)\\ \; & =\frac{\sum_{s=0}^{S}\sum_{t=0}^{T}\left(K_{s}^{\prime },K^{\prime t}\right)}{\sqrt{ST}}\cdot e^{j2\pi (\frac{Y_{S}K_{s}^{\prime }}{S}+\frac{Y^{T}\cdot K^{\prime t}}{T})}, \end{array}$$

where $Y_{S}$ represents the eigenvalues on the discrete spatial coordinates in the frequency domain, and $Y^{T}$ denotes the eigenvalues on the discrete time coordinates in the frequency domain.

**Figure 5:**
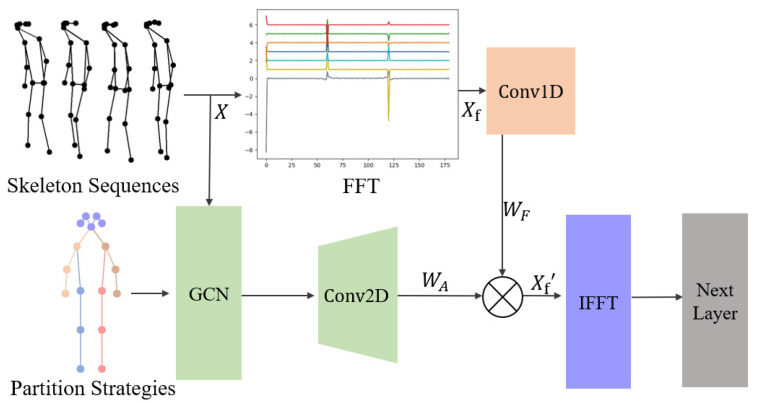
This figure illustrates the architecture of the F-GCN Network. The F-GCN operates on skeletal sequences and specified adjacency matrixes into consideration. Each intermediate variable in the calculation process is labeled, and the computation steps can be obtained by referring to Equation (3) through Equation (5).

The F-GCN enhances gait analysis by utilizing frequency domain weighting alongside data restoration techniques to optimize the process. This method effectively counters the computational distortions that arise when predicting human skeletal points through algorithms. F-GCN harnesses the intrinsic periodicity of human walking patterns by applying a frequency domain weighting tactic, thus capturing essential gait attributes with high precision. In addition, the network incorporates spatial data by preserving the original spatial characteristics within the frequency domain weighting phase. F-GCN’s integrated approach ensures a comprehensive feature representation, which embraces both the frequency and spatial dimensions, culminating in improved gait analysis performance.The detailed structure of the F-GCN is shown as Figure 5.

### 3.3. Frequency Pyramid Graph Convolutional Network

**Figure 6:**
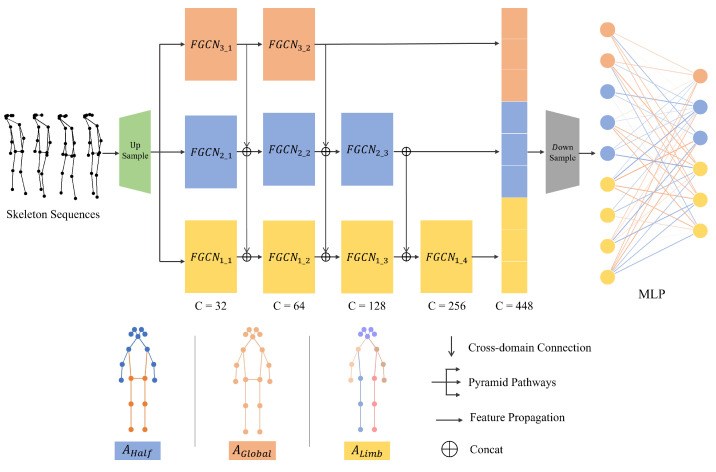
Figure of the FP-GCN Network Structure. This module integrating a multi-scale approach, featuring global, half, and limb pathways, along with cross-domain connections for effective information exchange.

This work builds upon the success of the Frequency Graph Convolutional Network (F-GCN) by introducing the Frequency Pyramid Graph Convolutional Network (FP-GCN) to further refine human gait analysis. Inspired by the SlowFast network’s [20] effective use of multiple pathways, FP-GCN takes a multi-scale approach to feature extraction. As shown in Figure 6, the FP-GCN utilizes three distinct pathways that analyze gait data at different granularities:

Global Pathway (Full Body): This pathway analyzes data from all joints simultaneously. It excels at capturing subtle motion correlations between various body parts, which might be undetectable by the human eye. However, this comprehensive analysis comes at a cost - it requires more computational resources and operates at slower speeds.

Half Pathway (Upper Lower Body): This pathway strikes a balance between detail and efficiency. It operates at a medium speed and computational load by analyzing the upper and lower halves of the body separately. This allows FP-GCN to identify interdependencies in motion between these two major sections, providing valuable insights into potential gait imbalances.

Limb Pathway: This pathway focuses on the most granular level of detail, extracting features specifically from the limbs and torso. As the fastest and least computationally demanding pathway, it’s ideal for identifying nuanced movements like high leg lifts or circular gait patterns often associated with conditions like hemiplegia.

The formula for eath pathway can be rewritten as:

(6) $$\begin{array}{cc} f_{\left(ph,i\right)} & =\sigma (W_{ph}^{FGCN}*x_{i}), \end{array}$$

where $f_{\left(ph,i\right)}$ represents the features of the ph-th Pathway for the *i*-th node; σ(·) represents the ReLU activation function; $W_{ph\_i}^{FGCN}$ represents the F-GCN weight specific to the ph-th Pathway; and the $x_{i}$ represents the input of *i*-th node.

This multi-scale approach allows FP-GCN to capture both the static body posture and the dynamic movement patterns of the torso, joints, and limbs. By analyzing these aspects simultaneously, FP-GCN extracts richer spatial-temporal joint dependencies, leading to superior performance in gait analysis tasks.

While the multi-scale pathways provide a comprehensive view of gait data, FP-GCN further enhances feature learning through a cross-domain connection mechanism. Inspired by advancements in U-Net++ [21] and DeepLab [22], this mechanism facilitates efficient communication of features across different spatial scales.

Shallow convolutional layers, particularly those in the Limb Pathway, excel at capturing detailed spatial information about limb movements. However, these features lack broader context about the entire body motion. Cross-domain connections address this by strategically integrating information from the Global Pathway, which analyzes the full body, into the Half and Limb Pathways during feature propagation. This essentially injects high-level semantic understanding into the lower-level, detailed features. The formula for Cross Domain Connection can be written as:

(7) $$\begin{array}{c} f_{\left(ph,i\right)}^{m}=W_{\left(ph,i-1\right)}*f_{\left(ph,i-1\right)}⊕W_{\left(ph-1,i\right)}*f_{\left(ph-1,i\right)}, \end{array}$$

where $f_{\left(ph,i\right)}^{m}$ represents the fusion features of the i+1 node of the *m*-th scale; $W_{\left(ph,i\right)}$ represents the weight associated with the *p*-th Pathway for the *i*-th node; and $f_{\left(ph,i\right)}$ represents the features of the *p*-th Pathway for the *i*-th node; ⊕ represents the concatenate operation.

FP-GCN’s multi-scale architecture excels at gait analysis due to its ability to capture data at different levels of detail (both broad body movements and intricate joint movements). This allows FP-GCN to recognize subtle gait abnormalities even in situations where parts of the body are obscured. This is particularly important in gait analysis, where identifying minute variations in gait cycles is crucial for diagnosis. Additionally, FP-GCN’s diverse pathways demonstrate remarkable adaptability to various walking patterns and speeds, highlighting its versatility in handling a wide range of motion information.

## 4. Experiments & Results

Both the GIST dataset [17] and our dataset, collected from various web resources, were selected for a comparative experiment with our work and existing methods. Datasets with limited samples, like the MMGS [23] (containing only 475 samples), were excluded to avoid potential overfitting in deep learning models due to insufficient training data.

**Figure 7:**
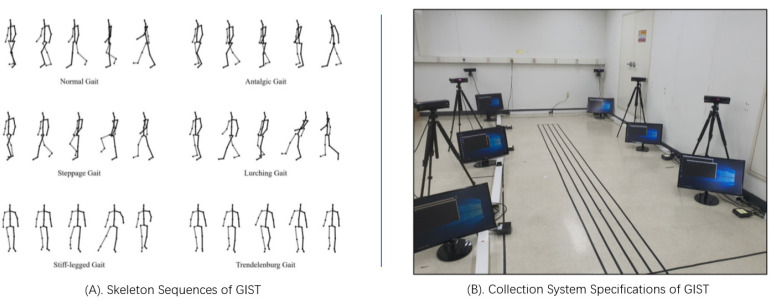
Illustration of data and the collection methods of GIST Dataset [17].

As shown in Figure 7, the GIST dataset comprises 6 gait pathologies from 10 subjects, with 120 frames per subject. This translates to a total of 7200 subjects captured by 6 Kinect V2 RGB-D cameras from 6 different viewpoints. For each subject, 25 skeleton points are extracted from the body using the Microsoft SDK, including the x, y, and z coordinates of each point. The captured gait sequences are classified into 6 categories: Normal, Stiff, Antalgic, Lurching (Wadding), Steppage, and Trendelenburg.

Since public datasets are typically collected using static RGB-D cameras, we sought to challenge our model’s generalizability by incorporating data from various devices. To this end, we established our own pathological gait dataset encompassing 9 gait types: 8 pathological and 1 normal.

**Figure 8:**
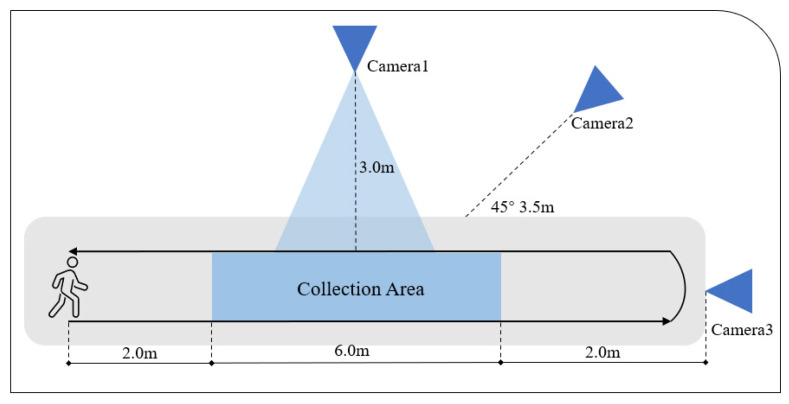
Illustration of data and the collection methods of our Dataset in hospital. Camera 1 videos side-view images from 3 m, Camera 2 captured oblique side-view videos from 3.5 m at a 45-degree angle, and Camera 3 captured frontal videos. The filming area covered six meters.

To enhance the experimental credibility and incorporate real-world scenarios, we collaborated with a neurology department at a Chinese hospital. With informed patient consent, we recorded pathological gait data from 8 real patients: 1 rehabilitated patient with normal gait, 2 patients with wadding gait, 1 patient with ataxia, 2 patients with hemiplegic gait, and 2 patients with steppage gait. As shown in Figure 8, the data were collected within hospital wards, where patients ambulated along a 10-m corridor unaided by assistive devices. To capture their gait characteristics comprehensively, we strategically positioned optical cameras at three distinct angles: lateral, oblique, and anterior. Each video segment endured for approximately 30 s, providing ample footage to analyze the walking patterns of patients. Although some patients may have encountered challenges in maintaining their walking direction, which led to minor adjustments in camera positioning, the overall setup proficiently recorded extensive gait data.

For the normal gait data, we leveraged the CASIA-B dataset [24]. We selected 13 healthy subjects, each contributing 11 video sequences captured from 11 different viewpoints, resulting in a total of 143 normal gait videos. To further enrich the pathological gait section of our dataset, we collected 74 gait videos from various online sources (e.g., YouTube) encompassing 60 individual subjects. These videos, captured using diverse devices, exhibit varying quality levels, some falling below the GIST dataset’s standards, which contributes to the dataset’s challenging.

**Figure 9:**
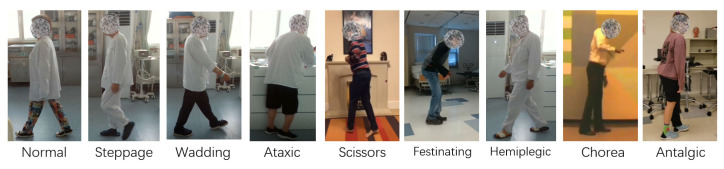
Some examples of our Gait dataset. This dataset contained 81 individuals with 9 kinds of gait, 8 pathological and 1 normal.

Considering the substantial training data requirements for both our model and ST-GCN, we employed data augmentation techniques to expand the number of pathological gait videos. Included mirroring, channel flipping, adding noise, and random cropping. Ultimately, we constructed the pathological gait section of our dataset with a total of 1644 videos. Figure 9 shows some examples of our dataset.

### 4.1. Training and Evaluation Metric

During the training process, the dataset was divided into three sets: the training set, the validation set, and the test set, with a ratio of 8:1:1. We employed SGD (Stochastic Gradient Descent) as the optimizer, with an initial learning rate of 1 × $10^{-4}$. we revise this, please confirm. This research utilized the label smoothing cross-entropy loss function. The decision to employ this technique was based on the hypothesis that pathological gaits share intrinsic links, and label smoothing is presumed to enhance the generalization capabilities of the resulting model. Each model was trained for 200 epochs with a batch size set to 64. The entire training process took approximately 36 h on a desktop PC equipped with an AMD^®^ Ryzen^TM^ 9 5950X CPU (Advanced Micro Devices, Incorporated, Sunnyvale, CA, USA), 64 GB of memory, and a NVIDIA^®^ GeForce^TM^ RTX3090 GPU (NVIDIA Corporation, Santa Clara, CA, USA) under CUDA 12.0 and PyTorch 1.13 conditions.

As for the evaluation metric, the classification accuracy was obtained by calculating the average accuracy as Equation (8).

(8) $$Accuracy=\frac{TP+TN}{TP+FP+TN+FN}.$$

Here, TP means the true positives; FP means the false positives; TN means the true negatives and FN means false negatives.

### 4.2. Experiment on GIST Dataset

To evaluate the effectiveness of our proposed method, we compared it with results obtained from various deep learning-based classifiers as reported in previous researches [15,17,25,26]. These models were used to classify one normal gait and five pathological gaits in the GIST dataset. The network-based models, including GRU, LSTM, basic RNN, and DNN, each have a four-layer structure with 125 hidden neurons per layer. The CNN classifier uses a 1D convolutional kernel with a size of 32 and three channels at the input layer. The output from the CNN is propagated by fully connected layers, consisting of a five-layer structure with 125 hidden neurons per layer. Our method was test in GIST dataset included 720 subjects (10 people × 6 gaits × 12 instances). Detailed test environment is the same with Section 4.1.

Table 1 shows the comparative experiment among the proposed model and the other 8 conventional schemes (as DNN, CNN, RNN, LSTM, GRU, CNN-LSTM, ST-GCN, AGS-GCN and Multiple-input ST-GCN) on the GIST Dataset.

The proposed method achieved the best performance as its recognition accuracy was up to 98.78%. That is because both the global spatial and temporal attention networks were introduced into the GCN. With the adaptive trainable weights applied in the GSA-GCN network, instead of compute the motion features from pre-defined regions, the proposed algorithm could automatically focus on meaningful human skeleton regions according to the training dataset. While the FP-GCN module could help the proposed to capture more detailed micro-motion of a patient and improve the accuracy of proposed method.

### 4.3. Experiment on Our Dataset

Besides the public dataset, another comparative experiment was conducted between the proposed method and ST-GCN [26] algorithm whose performance was similar to the proposed work on the GIST dataset. Other works like Multiple-input ST-GCN [17] and AGS-GCN [15] were not selected for this experiment due to the lack of their source code. The following Table 2 summarizes the composition of our dataset, detailing the number of subjects and video sequences for each gait. Our method was tested on a subset of this dataset consisting of 166 subjects (10% of the total). The detailed test environment is the same with Section 4.1.

Table 3 shows the detailed 9 kinds of gaits contained in our dataset as well as the performance of all compared methods. The proposed method was superior to ST-GCN on almost all the pathological gait data and its mean recognition accuracy was 96.54% which was 4.64% higher than that of ST-GCN. Compared with the experiment on the GIST dataset, the performance of ST-GCN has been degraded due to the following reasons: (1) the videos in our dataset were captured by the various recorders and conditions, where the background is cluttered and motion noise (which the ST-GCN may suffer from) was also included; (2) the cycles of each pathological gait vary greatly from each other, and the fixed temporal step in ST-GCN had limited its recognition accuracy; (3) since the data dimension of our dataset is 2D, it contains less information than the GIST, and correspondinglly the performance of ST-GCN was degraded. As for the festinating gait, ST-GCN was a little superior (only 0.1%) to the proposed method, where such gait contains no outstanding features and the performance of the two algorithms was quite similar to each other.

### 4.4. Ablation Study

To comprehensively evaluate the effectiveness of FP-GCN, we conducted ablation studies to investigate the impact of different components and design choices on its performance. Specifically, we examined the following variations:

F-GCN (Frequency Graph Convolutional Network): Focuses on capturing temporal patterns through spectral decomposition, highlighting its importance in gait analysis.

P-GCN (Pyramidal Graph Convolutional Network): Emphasizes spatial feature extraction by analyzing inter-sensor dependencies with pyramidal feature extraction.

FP-GCN (Frequency-Pyramidal Graph Convolutional Network): Combines the strengths of F-GCN and P-GCN, utilizing both spectral decomposition and pyramidal feature extraction for comprehensive analysis.

The results of the ablation experiments conducted on the GIST gait classification dataset (refer to Table 4) provide valuable insights. F-GCN (Frequency Graph Convolutional Network) achieves an accuracy of 96.75%, emphasizing the importance of temporal pattern capture through spectral decomposition. P-GCN, focusing on spatial feature extraction with pyramidal features, achieves a slightly lower accuracy of 95.18%. This suggests that while spatial features are valuable, incorporating temporal information is crucial. The combined model, FP-GCN, outperforms both individual models with an impressive accuracy of 98.78%, representing a 3.6% improvement. This significant improvement highlights the effectiveness of integrating spectral decomposition and pyramidal feature extraction for comprehensive gait analysis. This approach has the potential to significantly advance healthcare applications by enabling more accurate gait-based diagnoses.

## 5. Discussion

However, our method is subject to certain limitations. Experiments have shown that frequency domain processing may decrease accuracy when dealing with the unpredictable tremors typical of Parkinson’s disease patients. Furthermore, the reliance on pose estimation algorithms for pre-processing is a challenge. These algorithms require high-quality video to get skeleton sequences. During processing, these algorithms are susceptible to external noise, such as self-occlusion, which can result in instability or loss of skeleton points, affecting the accuracy of the algorithms.Finally, rigorous patient privacy regulations present significant challenges for data acquisition, hindering the accurate assessment of disease severity.

We consider our work could be further improved in the following ways:

Expanding the pathological gait dataset: To discover the full potential of pathological gait analysis in clinical settings, requires capturing patient data throughout their treatment under the guidance of medical experts. Including information on symptom severity, treatment plans, and long-term outcomes will yield deeper insights into the progression of gait disorders and treatment efficacy. This enriched data will ultimately pave the way for more effective diagnostic and therapeutic strategies to improve patient outcomes.

Considering 3D skeletal sequences as a solution: This approach facilitates observation from multiple perspectives, enabling a more comprehensive capture of subtle variations and details in patient gait. Consequently, the model gains enhanced insight into patient gait patterns, providing a more accurate foundation for pathological gait classification and diagnosis.

Establishing a musculoskeletal motion model of humans: Gait analysis enables the exploration of the relationship between specific types of pathological gait and the affected muscles or tissues, aiding in identifying the precise location of pathology within specific muscles and tissues.

## 6. Conclusions

This paper introduced the Frequency Pyramid Graph Convolutional Network (FP-GCN), a novel approach that enhances spatial feature extraction and complements temporal analysis in pathological gait classification. FP-GCN leverages spectral decomposition to capture gait data across different time scales, enabling the detection of rhythmic patterns and gait velocity variations. This facilitates a more comprehensive analysis of temporal features compared to existing methods. Experimental results on various datasets showcase the effectiveness of FP-GCN, achieving outstanding accuracy of 98.78% on public dataset and 96.54% on proprietary dataset.

## Figures and Tables

**Table 1:** Comparison with other models on GIST Dataset [17]. Here, the proposed method achieved the best performance by utilizing all the related arthrosis for pathological gait recognition.

Model	Train Accuracy (%)
DNN	85.86
CNN	86.65
RNN	86.63
LSTM	87.25
CNN-LSTM [25]	90.10
GRU	90.13
ST-GCN [26]	94.50
AGS-GCN [15]	94.56
Multiple-input ST-GCN [27]	98.34
Our Model (FP-GCN)	98.78

**Table 2:** Summary of our dataset: It consists of 68 subjects, resulting in 1644 videos. The data is categorized into nine gait classes: Normal, Steppage, Wadding, Ataxic, Scissors, Festinating, Hemiplegic, Chorea, and Antalgic.

Class	Subject	Video (Augmented)
Normal	13 Web + 1 Real	180
Steppage	8 Web + 2 Real	200
Wadding	8 Web + 2 Real	200
Ataxic	6 Web + 1 Real	180
Scissors	5 Web	180
Festinating	10 Web	180
Hemiplegic	10 Web + 2 Real	220
Chorea	6 Web	144
Antalgic	8 Web	168
Total	82	1644

**Table 3:** Comparative results of different models on our dataset (%). The recognition accuracy of our models is much better than the conventional ST-GCN method due to their observation over the arthrosis across the entire human body.

Class	ST-GCN	Our Model
Normal	89.3	94.6
Steppage	95.6	96.4
Wadding	88.2	97.5
Ataxic	88.9	92.5
Scissors	82.6	94.5
Festinating	94.7	94.6
Hemiplegic	95.2	96.7
Chorea	94.0	96.5
Antalgic	94.7	96.2
Mean Accuracy	91.92	96.54

**Table 4:** Results of Ablation Experiments on GIST Dataset.

Model	Accuracy (%)
F-GCN	96.75
P-GCN	95.18
FP-GCN	98.78

## Data Availability

The data used to support the findings of this study are available from the corresponding author upon request. The data are not publicly available due to personal information and privacy about the data.

## References

[1] Dingwell J.B., Cusumano J.P. (2000). Nonlinear time series analysis of normal and pathological human walking. Chaos Interdiscip. J. Nonlinear Sci..

[2] Zhang Y., Ogunbona P.O., Li W., Munro B., Wallace G.G. Pathological Gait Detection of Parkinson’s Disease Using Sparse Representation. Proceedings of the 2013 International Conference on Digital Image Computing: Techniques and Applications (DICTA).

[3] Guo G., Guffey K., Chen W., Pergami P. (2017). Classification of Normal and Pathological Gait in Young Children Based on Foot Pressure Data. Neuroinformatics.

[4] Tao W., Liu T., Zheng R., Feng H. (2012). Gait Analysis Using Wearable Sensors. Sensors.

[5] Rentz C., Far M.S., Boltes M., Schnitzler A., Amunts K., Dukart J., Minnerop M. (2022). System Comparison for Gait and Balance Monitoring Used for the Evaluation of a Home-Based Training. Sensors.

[6] Ivanov K., Mei Z., Lubich L., Guo N., Xile D., Zhao Z., Omisore O.M., Ho D., Wang L., Yu H., Liu J., Liu L., Ju Z., Liu Y., Zhou D. (2019). Design of a Sensor Insole for Gait Analysis. Intelligent Robotics and Applications.

[7] Schlagenhauf F., Sreeram S., Singhose W. (2018). Comparison of kinect and vicon motion capture of upper-body joint angle tracking. Proceedings of the 2018 IEEE 14th International Conference on Control and Automation (ICCA).

[8] Albuquerque P., Verlekar T.T., Correia P.L., Soares L.D. (2021). A Spatiotemporal Deep Learning Approach for Automatic Pathological Gait Classification. Sensors.

[9] Ortells J., Herrero-Ezquerro M.T., Mollineda R.A. (2018). Vision-based gait impairment analysis for aided diagnosis. Med. Biol. Eng. Comput..

[10] Loureiro J., Correia P.L. Using a Skeleton Gait Energy Image for Pathological Gait Classification. Proceedings of the 2020 15th IEEE International Conference on Automatic Face and Gesture Recognition (FG 2020).

[11] Nghiem A.T., Auvinet E., Multon F., Meunier J. Contactless abnormal gait detection. Proceedings of the 2011 Annual International Conference of the IEEE Engineering in Medicine and Biology Society.

[12] Bei S., Zhen Z., Xing Z., Taocheng L., Qin L. (2018). Movement Disorder Detection via Adaptively Fused Gait Analysis Based on Kinect Sensors. IEEE Sens. J..

[13] Gu X., Guo Y., Yang G.Z., Lo B. (2022). Cross-Domain Self-Supervised Complete Geometric Representation Learning for Real-Scanned Point Cloud Based Pathological Gait Analysis. IEEE J. Biomed. Health Inform..

[14] Zeng Q., Liu P., Bai Y., Yu H., Sun X., Han J., Wu J., Yu N. SlowFast GCN Network for Quantification of Parkinsonian Gait Using 2D Videos. Proceedings of the 2022 12th International Conference on CYBER Technology in Automation, Control, and Intelligent Systems (CYBER).

[15] Tian H., Ma X., Wu H., Li Y. (2022). Skeleton-based abnormal gait recognition with spatio-temporal attention enhanced gait-structural graph convolutional networks. Neurocomputing.

[16] Jun K., Oh S., Lee S., Lee D.W., Kim M.S. Automatic pathological gait recognition by a mobile robot using ultrawideband-based localization and a depth camera. Proceedings of the 2022 31st IEEE International Conference on Robot and Human Interactive Communication (RO-MAN).

[17] Jun K., Lee Y., Lee S., Lee D.W., Kim M.S. (2020). Pathological Gait Classification Using Kinect v2 and Gated Recurrent Neural Networks. IEEE Access.

[18] Dingenen B., Staes F.F., Santermans L., Steurs L., Eerdekens M., Geentjens J., Peers K.H.E., Thysen M., Deschamps K. (2018). Are two-dimensional measured frontal plane angles related to three-dimensional measured kinematic profiles during running?. Phys. Ther. Sport.

[19] Stenum J., Rossi C., Roemmich R.T. (2021). Two-dimensional video-based analysis of human gait using pose estimation. PLoS Comput. Biol..

[20] Feichtenhofer C., Fan H., Malik J., He K. Slowfast networks for video recognition. Proceedings of the IEEE/CVF International Conference on Computer Vision.

[21] Zhou Z., Rahman Siddiquee M.M., Tajbakhsh N., Liang J. (2018). Unet++: A nested u-net architecture for medical image segmentation. Proceedings of the Deep Learning in Medical Image Analysis and Multimodal Learning for Clinical Decision Support: 4th International Workshop, DLMIA 2018, and 8th International Workshop, ML-CDS 2018, Held in Conjunction with MICCAI 2018.

[22] Yu F., Wang D., Shelhamer E., Darrell T. Deep layer aggregation. Proceedings of the IEEE Conference on Computer Vision and Pattern Recognition.

[23] Khokhlova M., Migniot C., Morozov A., Sushkova O., Dipanda A. (2019). Normal and pathological gait classification LSTM model. Artif. Intell. Med..

[24] Yu S., Tan D., Tan T. (2006). A framework for evaluating the effect of view angle, clothing and carrying condition on gait recognition. Proceedings of the 18th International Conference on Pattern Recognition (ICPR’06).

[25] Sadeghzadehyazdi N., Batabyal T., Acton S.T. (2021). Modeling spatiotemporal patterns of gait anomaly with a CNN-LSTM deep neural network. Expert Syst. Appl..

[26] Yan S., Xiong Y., Lin D. Spatial temporal graph convolutional networks for skeleton-based action recognition. Proceedings of the Thirty-Second AAAI Conference on Artificial Intelligence.

[27] Kim J., Seo H., Naseem M.T., Lee C.S. (2022). Pathological-Gait Recognition Using Spatiotemporal Graph Convolutional Networks and Attention Model. Sensors.

